# Rapid transition of a preclinical health systems science and social justice course to remote learning in the time of coronavirus

**DOI:** 10.1080/10872981.2020.1812225

**Published:** 2020-08-21

**Authors:** Megha Garg, Archna Eniasivam, Jason Satterfield, Betsy Norton, Elizabeth Austin, Daniel Dohan

**Affiliations:** a Department of Medicine, University of California San Francisco and San Francisco Veterans Affairs Medical Center, San Francisco, CA, USA; b Departments of Medicine and Pediatrics, University of California San Francisco, San Francisco, CA, USA; c Division of General Internal Medicine, University of California San Francisco, San Francisco, CA, USA; d School of Medicine, University of California San Francisco, San Francisco, CA, USA; e Philip R. Lee Institute for Health Policy Studies, University of California San Francisco, San Francisco, CA, USA

**Keywords:** Remote learning, coronavirus, health systems science, social justice, preclinical curriculum

## Abstract

As the coronavirus pandemic started, we rapidly transitioned a preclinical social justice and health systems sciences course at our medical school to asynchronous, remote learning. We describe processes, curricular innovations, and lessons learned. Small groups were converted into independent learning modules and lectures were given live via videoconferencing technology. We started with a simplified approach and then built technological capabilities over time. Current events were incorporated into curriculum and assessment. Our course ran from 16 March–3 April 2020 for the 155-person first-year class. Student attendance for optional, synchronous remote sessions was higher than in-person attendance in previous years. Completion rates for assignments were high but with minimal student collaboration. Faculty office hours were underutilized. Focus group and formal evaluations were largely positive, with numerical ratings for quality of the course and faculty teaching higher than the 2 years prior. Student engagement with social justice topics in aremote format was successful through modifications to small groups and lecture structure. Students, faculty, and administrative staff appreciated the consistency of session format throughout the course. Students exam performance was similar to prior years. Attention should be paid to what can be learned via self-study as opposed to small group learning. Better methods of soliciting real-time student feedback, and encouraging engagement with each other and with faculty in aremote environment are needed.

## Introduction

The coronavirus pandemic (COVID-19) changed the world in a matter of weeks. To comply with social distancing and shelter-in-place mandates, medical schools around the country urgently transitioned to remote learning without significant time for preparation[1].

The University of California San Francisco (UCSF) School of Medicine’s innovative Bridges curriculum includes a three-week required course, Health and Society (H&S), focused on health systems science (HSS) and social justice [2,3]. H&S includes topics of health policy and systems, advocacy, and health determinants and disparities. Offered in the first year of medical school, the objectives, content, and style of H&S differ substantially from the biomedical science curriculum. Although schools are already embracing virtual learning as essential for the future of medical education[4], COVID-19 required transitioning this unique content into a wholly virtual format in 1 week. We share processes, curricular innovations, and lessons learned as we rapidly transitioned our course to a remote learning format.

## Methods

The original H&S course curriculum included large group lectures and panel discussions, small groups led by facilitators, and independent assignments such as videos, essays, and a book club. Our in-person teaching methods followed exemplar courses that use a mix of teaching strategies, often in small group formats, to maximize student engagement and deepen self-reflection[5]. Remote formats for topics like structural racism, power and privilege, and health disparities were considered less than ideal and potentially harmful for students.

Additionally, institutional and city government policies were evolving quickly, creating uncertainty in parameters for teaching. UCSF leadership issued an initial communication advising H&S course directors to consider alternatives to large group lectures to comply with physical distancing requirements on 6 March, 10 days before the course start date. On 11 March, the school announced cancellation of all in-person learning effective 16 March, the first day of H&S. Furthermore, San Francisco anticipated a surge of critically ill COVID-19 patients; most H&S teaching faculty are clinicians and their availability was unpredictable. Students were processing the world’s events while experiencing a complete overhaul of their education, and many were transitioning to new locations, often in different time zones, anticipating a remote education for the foreseeable future. San Francisco issued its mandatory shelter-in-place order on the first day of H&S, requiring residents to stay home and leave only for essential needs to prevent community transmission of coronavirus, and forcing all subsequent teaching and course coordination online.

The abrupt changes left limited time to make decisions or test technological capabilities. Furthermore, we did not have the time to train facilitators in unfamiliar remote learning or virtual classroom requirements, a formidable challenge even when time is not a limitation [6,7]. Our approach followed an educational roadmap outlined by Tolsgaard and colleagues including triage of curricular content and how best to enact changes[8]. We ultimately implemented a technologically simple yet robust curriculum that could preserve core course objectives at the outset and build upon that during the course. We anticipated particular challenges around experiential small groups that addressed content that could potentially trigger strong or harmful emotional responses, based on our previous in-person experiences during which recognizing non-verbal communication and ad hoc post-session debriefs with faculty had been necessary to process the material. In an online environment, it would be harder to discern body language or approach individuals during breaks or after a session to address difficult interactions. Table 1 outlines the process changes that were made for each component of the course.

**Table 1.** Process changes for rapid transition to remote learning.

Original Design	Technology Changes	Curricular Changes
SMALL GROUPS		
Student discussion groups of 12–14 with a trained facilitator In-person facilitation using a student guide and separate facilitator guide Content could include emotionally charged and triggering topics such as power and privilege	Converted to asynchronous, individual learning modules with no facilitator Students were provided a modified student guide with facilitator guides provided to check learning	Encouraged optional remote group work with students Role plays eliminated Preamble for remote learning norms/expectations, including ‘do not meet in person’ mandate Reflections and assignments designed to create an emotionally valid and memorable experience Completion assignments timed to be due near original timing of small group to help with pacing
LECTURES		
In-person large group didactic lectures with optional attendance Recorded for asynchronous viewing option Limited but some opportunities to ask questions and interact with lecturer	Option to view synchronously as faculty member presented remotely with interaction via chat Recorded for asynchronous viewing option Technology testing ahead of time for each panelist/faculty member	Have a different faculty member serve as ‘chat master’ for the lecture to manage questions and promote student engagement Learner questions were selected and read by the chat master Lecturers were instructed to reduce quantity of slides and create openings for discussion
PANELS		
In-person panels with mandatory attendance in large group setting Often with guests including patients Not recorded, no asynchronous viewing option	Patient panels canceled, all others converted to synchronous, remote panels All panelists on video at all times With panelist permission, recorded for optional asynchronous viewing	Active moderation is necessary to manage discussion and transitions between speakers Email questions beforehand to panelists Suggest time limits for answers Follow up forum posts with chat questions that were not answered, after posing questions to panelists via email

## Small groups

On 12 March, we, as course leadership, made the difficult decision to convert all small groups to individual learning modules that would not require a facilitator and could be completed by students asynchronously, from different times and locations. We initially considered cancelling small groups only for week 1 of the course while preparing for remote facilitation of high-priority groups during weeks 2–3. This approach was rejected due to concerns about technological feasibility, small group leader availability, and potential for negative experiences for students engaging with emotionally charged topics in a digital setting. We believed that independent learning that included experiential and applied exercises provided the best way to ensure a consistent, learner centered approach, especially important for HSS and social justice topics[9]. We thus rewrote our small group guides from an in-person to an online format driven by established best practices for the creation of digital learning objects and e-learning adaptations [10–13].

In general, the new self-study ‘small groups’ included knowledge acquisition, reflection, discussion, and application to a current and/or clinical issue, followed by some form of reporting back. However, conversion to a self-study format presented unique challenges. For example, an advocacy small group originally included a 30-minute role play simulating a legislative visit. We cut this exercise and expanded the opinion-writing section, allotting time to write an opinion piece regarding COVID-19 and optionally submit for publication. This assignment led to five students successfully publishing their work in local and national publications, including in the *New York Times* [14–16].

We significantly modified a power and privilege small group intended to delve into topics such as white fragility and white silence [17,18] that could potentially trigger defensiveness or vulnerability. We converted this into a self-reflection exercise for students to write about their own silence, identities, and privileges, which resulted in powerful essays that students voluntarily shared in a moderated online environment. In general, the content and format of the small group drove the digital conversion strategies, with knowledge acquisition being the simplest to convert. Experiential or discussion-based learning required new reflection exercises that could be completed alone in an authentic way.

We also released the original facilitator guides and discussion notes to students to check their learning. We recognized that this may result in inappropriate dissemination for future students, but decided that student support was paramount. We asked that students view the guides only after completion of independent learning to ensure they understood the key points. We offered remote office hours regularly to allow for real-time clarification of the material. Accountability was sustained by the continued use of the students’ online learning portal, online forum discussion posts, and tracking of assignment completion by course administrators.

## Lectures and panel discussions

Compared to the challenge of small groups, moving lectures online seemed relatively straightforward. To reduce the burden on clinical faculty and technology, we initially decided to use pre-recorded online lectures to cover core content on health insurance and health systems. However, H&S lecturers were concerned about this plan, feeling that a live lecture was important for delivering key learning points and responding to the institutional and health systems implications of COVID-19. Therefore, we held remote, synchronous (live) lectures using video conferencing technology. Live lecture attendance was made optional and all sessions were recorded to allow asynchronous access. Students could interact with course faculty via live chat functions through which they could type comments and questions during the lecture. Lecturers were asked to streamline their content to allow time for discussion. Course leadership managed the typed chat conversation during lectures and presented student questions to the lecturers. The unanticipated benefit of this format was that students could share related references and answer each other’s questions, elevating the level of engagement during the lecture. Moreover, more reticent students could privately chat with faculty if they were uncomfortable posting publicly.

The initial H&S lectures occurred remotely on 16 March– 1 day before mandated shelter-in-place – and were conducted from conference rooms that allowed faculty and staff to navigate the challenges in person while also maintaining necessary social distance. Based on this experience, we developed online training for all faculty lecturers. Faculty successfully delivered synchronous lectures from sheltered locations (typically their home) while maintaining a live audience similar to an in-person lecture.

## New content

We felt it was important to acknowledge COVID-19 during the course, as it was emotionally affecting students, faculty, and medical school leadership. Furthermore, given the focus of the course is health systems, determinants of health, and social justice, the public health crisis provided an opportunity to meaningfully apply course objectives. Table 2 describes innovations related to COVID-19 content.

**Table 2.** HSS/social justice-related curriculum innovations pertaining to COVID-19.

COVID-19 Curriculum Innovations for HSS and Social Justice Content

Introductory lecture on public health institutions and pandemic preparation
COVID-19 ‘on the ground’ panel discussions with frontline clinicians – topics included federal, state and local response to the pandemic, ethical considerations regarding limited resources, and emerging health disparities in the care of patients.
‘Theories of Justice’ ethics case about applying resource allocation considerations in the time of COVID-19
Physician Advocacy session assignment to write an op-ed to a local newspaper regarding COVID-19
Assessment assignments throughout the course modified to include coronavirus related case examples
For a panel on physician payment, panelists asked to comment on how COVID-19 impacted their daily practice and income

We added a new lecture on public health institutions and their approach to public health crises. Since a number of course faculty were on the front lines of COVID-19 clinical care, we also hosted two panel discussions – one at the beginning and one at the end of the course – focused on the front line experience of the pandemic. These panels were informal reflections with opportunities for students to ask questions about any aspect of the experience, and incorporated some best practices for engagement in remote learning, such as bringing in a personal connection and active participation via videoconference chat functions[19]. We also incorporated current event case examples into mandatory weekly assessment assignments.

## Technology

Technological capabilities were among the most significant considerations in our final course plan. Though our institution possessed a wide array of remote capabilities, much of the technology was untested, and the technology team was stretched over the educational transition occurring across the entire medical school. We also could not assume that all of our students were digital natives who were adept with new technologies[20], or that they had equal access to digital resources and accessibilities[6]. This influenced our short-term decision-making to focus on simpler formats such as large lectures and to minimize the technological complexities of managing multiple small group facilitators. Only after we successfully implemented our initial plan did we consider piloting more complicated technologies like small group discussion sections.

Once a plan was determined, the technology team and course administrators were instrumental in executing time-intensive technology testing meetings with each faculty member prior to their sessions. A technology team member also joined to support and troubleshoot during each synchronous session. Additionally, we relied on chat programs and phone texting for faculty to communicate outside of video conferencing during live sessions.

We also considered students’ access to technology, given that many left town to return home. We made individual allowances for travel time to ensure they would have consistent access to the internet to be able to meaningfully participate in the curriculum.

## Results

Our course ran from 16 March to 3 April 2020. Given the last-minute course conversion to remote learning, we were pleased with the positive response from the students. Student attendance at optional, synchronous remote lectures, measured by number of participants in the remote sessions, ranged from 25% to over 50% of the 155 first-year student class. This surpassed our experience of previous years’ in-person lecture attendance, though we did not take formal attendance at optional lectures in the past. Panel discussions, including the COVID-19 panels, had the highest attendance. Students also completed required assignments related to individual learning modules without difficulty. The exam was a remote, closed-book short essay format with few modifications to course objectives or exam length. It was given during a predetermined time using a secure browser, and students could ask questions via email to course directors. Students were reminded of the institution’s honor code and re-committed to work independently. All students completed the exam within the allotted time and performed similarly to previous years.

There were some opportunities to enhance the remote learning experience. When surveyed (upon turning in assignments online) about whether they optionally worked together via remote technologies, which we had encouraged, only 10% of students indicated that they worked with one or more students. This is an improvement opportunity to explore as we continue with remote learning. Another area for improvement is student feedback and interaction with faculty during the course. Faculty office hours via live video conferencing were offered almost every day of the course but were poorly attended.

An informal focus group with 10 student volunteer participants was conducted the week after the course ended without faculty present to gather feedback on the remote learning experience. Among the themes noted were appreciation for follow up forum posts for questions not answered during lectures and consistency of expectations throughout the course. Students preferred lectures in which Q&A was saved for the end of the lecture, and had mixed reviews of independent learning modules. Some observed that independent learning modules may work particularly well for social justice and HSS content because of the focus on reflecting and processing personal experiences, but may not work as well for biomedical science courses. Additionally, they requested clear guidelines of how to engage with faculty in the online format.

Formal course evaluations were largely positive and improved over the years prior. In accordance with our institution’s evaluation practice of surveying a subset of students for each course to mitigate survey fatigue, 51 students were randomly selected from the class of 155 students to complete mandatory course evaluations; 47 students completed the evaluations (92%). The ‘overall quality of the course’ rating was 3.6 out of 5 (1 = Poor, 2 = Fair, 3 = Good, 4 = Very Good, 5 = Excellent), up from 3.0 in 2018 academic year. In 2017 the rating was 3.0, but the question was optional with only 10 responses. Average course instructor rating for 2019–2020 was 4.6, up from 4.3 in 2018, and 4.4 in 2017.

## Discussion

COVID-19 created an opportunity to quickly innovate and discover the suitability of remote learning for a preclinical medical school course focused on HSS and social justice issues. Due to limited time for planning or training, and consideration of student accessibility to digital resources, we opted to maintain consistency of session format rather than institute intermittent course changes. Students, faculty, and administrative staff endorsed this approach, and formal course evaluations improved compared to previous years.

Our experience demonstrated the possibility for innovation in pedagogy that can come with technology specifically for social justice topics that are now emerging as core content in a modern medical education [12,21]. Even prior to the exigencies of the current pandemic, our course faced the unique challenges of teaching about social justice topics in medicine. In contrast to traditional medical school topics like pathology and anatomy, our content asks students to explore and share personal identities and biases, and confront injustices like racism, sexism, and xenophobia. These topics require extra support for students and faculty alike in an in person setting, so we had to be very thoughtful about how to engage students while also maintaining a safe learning environment.

Surprisingly, we felt that small groups that addressed difficult topics were well-received when converted to a self-study modality. Sessions worked well logistically without faculty facilitators, and students actively engaged in individual reflection. Social justice topics such as physician advocacy and power and privilege were especially successful in this format, reflected in the quality of assignments completed, student publications, and positive course evaluations. While the opportunity to discuss and learn from another remains important, the value of self-study exercises is highlighted by our experience. As a result, we plan to maintain robust individual learning opportunities blended with facilitated discussion in future years even when in-person teaching is possible.

The COVID-19 experience also forced us to pilot teaching modalities that we had not previously used. Not only were these new multimodal methods successful on their own, but they also freed up course time that in the future can be used to improve synchronous learning (both in-person and remote) through expanded training of small group facilitators and engaging students in the development of remote learning practices. Recognizing that there may be future similar interruptions to medical education, we continue to consider innovations that we will embed in the curriculum beyond this remote learning period[22].

Lectures and panel discussions, which translated directly to remote formats, offered increased opportunities to engage remote speakers and for student participation via chat functions and post-lecture communications. We discovered an unanticipated benefit of being able to measure attendance in the virtual setting automatically with the number of participants visible, which was not done for optional lectures in previous years. Finally, we cancelled patient panels due to the immediacy of transition to remote learning, but we anticipate that patient experiences will also work well remotely in the future and plan to ensure that patient narratives remain integrated into course learning.

Based on this experience, we identified a need for additional methods of soliciting real-time student feedback, and engaging each other and faculty. While faculty office hours were regularly offered, students did not utilize them often. They did email faculty with questions and feedback throughout the course, indicating that perhaps the completion of asynchronous work did not align with scheduled faculty office hours. We have also had low attendance during in-person faculty office hours in previous years, so this may not have been a reflection of the virtual learning environment but rather ongoing need to optimize the office hours structure. Although we built in some optional opportunities for faculty and peer engagement, the uptake was low, indicating that alternative methods of collaboration will be needed in future remote learning endeavors.
